# Transforming Nepal’s primary health care delivery system in global health era: addressing historical and current implementation challenges

**DOI:** 10.1186/s12992-022-00798-5

**Published:** 2022-01-31

**Authors:** Bipin Adhikari, Shiva Raj Mishra, Ryan Schwarz

**Affiliations:** 1 Nepal Community Health and Development Centre, Kathmandu, Nepal; 2 Mahidol-Oxford Tropical Medicine Research Unit, Faculty of Tropical Medicine, Mahidol University, Bangkok, Thailand; 3 Centre for Tropical Medicine and Global Health, Nuffield Department of Medicine, University of Oxford, Oxford, UK; 4 Nepal Development Centre, Bharatpur, Nepal; 5 Possible, New York, NY USA; 6 Brigham and Women’s Hospital, Department of Medicine, Division of Global Health Equity, Boston, MA USA; 7 Harvard Medical School, Department of Medicine, Boston, MA USA

**Keywords:** Primary health care, Health system, Rural health, Health human resources, Health insurance, Universal health coverage, Community health workers, Federalization, Sustainable development goals, Nepal

## Abstract

Nepal’s Primary Health Care (PHC) is aligned vertically with disease control programs at the core and a vast network of community health workers at the periphery. Aligning with the globalization of health and the factors affecting global burden of diseases, Nepal echoes the progressive increase in life expectancy, changes in diseases patterns, including the current impact of COVID-19. Nepal’s health system is also accommodating recent federalization, and thus it is critical to explore how the primary health care system is grappling the challenges amidst these changes. In this review, we conducted a narrative synthesis of literature to explore the challenges related to transformation of Nepal’s primary health care delivery system to meet the demands incurred by impact of globalization and recent federalization, covering following database: PubMED, Embase and Google Scholar. Of the 49 articles abstracted for full text review, 37 were included in the analyses. Existing theories were used for constructing the conceptual framework to explain the study findings. The results are divided into four themes. Additional searches were conducted to further support the narrative synthesis: a total of 46 articles were further included in the articulation of main findings. Transforming Nepal’s primary health care system requires a clear focus on following priority areas that include i) Revised efforts towards strengthening of community based primary health care units; ii) Adapting vertical programs to federal governance; iii) Reinforcing the health insurance scheme; and iv) Strengthening an existing network of community health workers and health human resources. This review discusses how these broad goals bear challenges and opportunities.

**Supplementary Information:**

The online version contains supplementary material available at 10.1186/s12992-022-00798-5.

## Key messages

- Globalization and health have integral impact and warrants a need to transform the health system which can be achieved through re-strengthening primary health care system by re-establishing community health care units and their functionalities (equipment/logistics and human resources).
- Immediate implementation of policies for clarity in distribution of responsibility, and accountability to all governance structure (federal, province and local) is critical..
- Strengthening of social health security system building on more research and evidence on costs of service delivery across the health sector; market costs; with improved quality of Universal Health Coverage (UHC) through multi-sectoral collaboration and public-private partnership needs prioritization.
- Improvement of quality of existing primary health care services based on community health worker assessment and improvement framework needs investments.

## Background

Nepal has made significant progress on health indicators over the past several decades [1]. The impressive achievement in health indicators was the result of globalization in health including economic development via-a-viz strengthening of primary (mostly peripheral) health care (PHC) health care (PHC) system particularly through investments to establish the health care infrastructure. PHC service in Nepal has been active since 1978 through a network of district and the distal network that reaches to the community served by health posts and sub-health posts. At the community level, nearly 50,000 Female Community Health Volunteers are mobilized throughout the country. Significant progress has been achieved by such a vast network of PHC in Nepal, a lot of which are reflected in millennium and sustainable development goal indicators [2]. Transforming health system to achieve millennium and sustainable development goal indicators also reflects how globalization has promoted health system to adopt these goals. The infant mortality rate declined by two fold from 78 deaths per 1000 live births in 1990 to 32 deaths per 1000 live births in 2016 and pregnancy related mortality rate declined by half from 543 deaths per 100,000 live births in 1989–1996 to 259 deaths in 2009–2016 [1, 2].

The progress over the decades is marred by socio-economic and geographic differences in access to health services. Despite showing an increment in institutional delivery rate from 18% in 2006, 39% in 2011 to 60% in 2016 for the recent birth, far less women from poor background utilize these services compared to those from richest wealth quintile in 2016. The 2016 Nepal Demographic Health Survey (NDHS) found only 36% of mothers from poorest wealth accessed institutional delivery compared to 92% of mothers from richest wealth quintile [3–5]. Similarly, birth assisted by skilled birth attendants showed increase from 19% in 2006, 36% in 2011 to 58% in 2016 however, only 34% of women from the lowest wealth quintile accessed SBA compared to 89% of women from richest wealth quintile. These disparities are expected to grow due to the impact of COVID-19 pandemic and may affect Nepal’s long-term aspirations in health (e.g. Sustainable Development Goals/SDGs), economy and development [3–6]. Globalization of the COVID-19 has clearly demonstrated how health is a global entity and political borders have little to no impact on restricting the spread of disease [7, 8]. Health system in low- and middle-income countries can suffer from additional burden due to inadequate preparedness, and weak primary health care system that can ultimately increase morbidity and mortality [9, 10]. Achieving the United Nation's (UN's) Sustainable Development Goals (SDG3: Ensure healthy lives and promote well-being for all at all ages) requires sheer attention in building primary health care [11].

The deficiencies and unmet targets in Nepal’s health system are often discussed and justified in terms of various challenges, a lot of which have been widely known (for e.g., geographical barriers) and are mostly accepted [12, 13]. However, there have not been a systematic attempt to explore what and how within the system and outside have affected the delivery of services, achievement of targets and goals. Although systematic review apparently can aid in gathering evidence around barriers and facilitators (factors) related to functioning of the health system; the complexity of the health system, services, and its operationalities pose unique challenges in comparing and consolidating these plethoric factors [14, 15]. Unlike how a systematic review (and meta-analysis) can forge evidence around certain clinical interventions (clinical trials), in complex social and programmatic interventions, their nature, particularly due to non-linearity and non-comparability, it demands a flexible and broader landscape for evidence synthesis [14, 16–20]. One such method adopted in this review is blending of relevant literature with the experiential and disciplinary expertise of the local social and cultural context of Nepal’s health system [21–23].

No previous study has examined implementation challenges in Nepal’s primary health care system (PHC) using a method that allows academic and experiential evidence [21–24]. Such methods can compensate the narrow and prescriptive outcome guided by a systematic review at the same time allows to include the comprehensive and historical evidence [14, 15]. The main objective of this article is to review the historical and current challenges and opportunities of Nepal’s PHC system in order to forge actionable recommendations for the future.

## Methods

This study utilized a narrative synthesis of literature, using a scoping review framework outlined by Arksey and O’Malley [25] which has been previously used in health system and policy context [26, 27]. Further incorporation of authors’ epistemic expertise allowed to compare and explore more commonalities (and differences) of the findings in the local social and cultural context [14, 15, 22, 23]. Our review framework incorporates the following phases: i) identifying the key research questions through an iterative review/discussion, ii) identifying the initial potential studies based on the discussion, iii) searching literature in major medical databases; iv) collating of data, synthesizing, and reporting of the findings, and v) discussion among experts and utilizing their feedback as a required steps in knowledge translation part of a scoping review methodology (see acknowledgements). Based on the series of these steps, we identified prominent themes that are categorized and presented as themes A, B, C, and D in corollary with the proposed framework (organization of main findings). The synthesized evidence was further mapped using a casual loop diagram (CLD) to show the complex interplay of factors in Nepal’s primary health care delivery system [26].

**Fig. 1:**
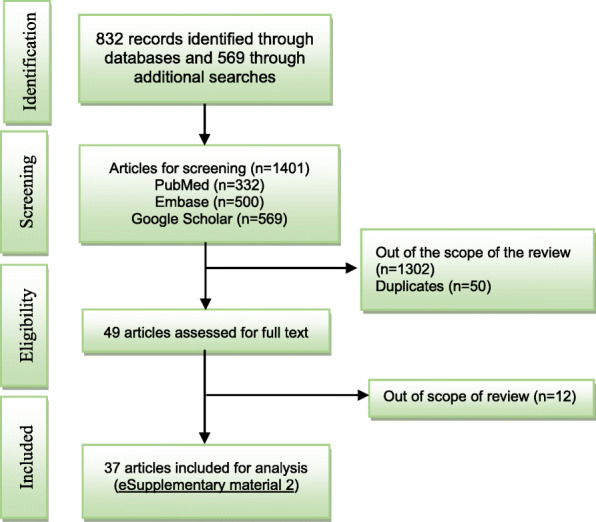
Flow diagram of the review process

We searched the medical databases (PubMed/Medline, Embase and Google Scholar) for scientific and grey literatures up until December 2020 using the following search terms in combination with Boolean operators “AND” and “OR”: “Nepal”, “Sustainable development goals”, “SDG”, “primary health care”, “PHC”, “health system”, “health system strengthening”, “health system integration”, “governance”, “accountability”, “health financing”, “human resources”, “supply chain”, “disease control”, “vertical programs”, “health outcomes”, “challenges”, “weaknesses”, “progress”, “success”, and “community health workers” (eSupplimentary material 1). Both peer reviewed publications, reports and webpages excerpts were considered for analyses. See the supplementary appendix for more information on search strategy. Further, the data on health expenditures, progress towards achieving millennium development and sustainable development goals were extracted from webpages of WHO, World Bank, and Nepal’s ministry of health and population. A total of 37 articles was selected in mutual consensus (BA, SRM) and abstracted in an excel sheet (Fig. 1, eSupplementary material 2).

### Organization of main findings

**Fig. 2:**
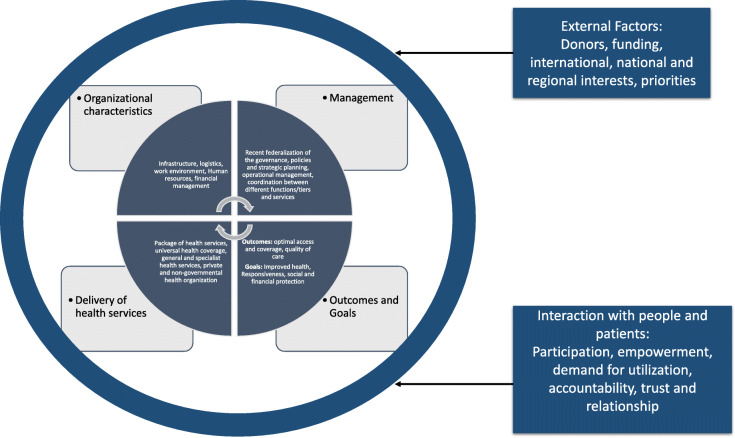
A conceptual framework showing the elements and aspects of health system as a normative vision on how a health system functions. This figure is adapted from health system framework based on van Olmen et al. [28] and WHO [29]

This narrative synthesis follows an iterative process [25] for exploration of relevant literature and synthesis of major findings using elements and components of health system and its functioning outlined by van Olmen et al. [28] and WHO [29] (Fig. 2). Specifically, themes that are relevant for health system in Nepal and supported by the framework were used. Final four themes were selected and are presented in the results sections that includes:

1. **Theme-A:** Historical development in primary health care delivery system
2. **Theme-B:** Transition of health system from unitary to federal system
3. **Theme-C:** Extending the risk pool for health
4. **Theme-D:** Primary health care system at crossroad

We followed additional steps to use experts’ feedback and utilized them in the synthesis of major findings. Such review process is unique for research aimed at untangling substantial, yet unique challenges facing health system delivery, and is further aimed at developing unbiased themes on major findings [30].

### Historical development in primary health care delivery system

From the Alma-Ata declaration of 1978, to the recent Astana Declaration, Nepal has continuously strived to reform PHC [31, 32]. These declarations and their commitment have engendered a shift in focus and transformed the way PHC worked in the past. Specifically, the shift from vertical governance to horizontal and community-based health program implementation bears a plethora of benefits including its contribution in community engagement in programs and health research [33–35]. Although the success through this declaration was not uniform around the globe, many countries including Nepal were able to achieve modest coverage in childhood immunization and access to water and sanitation particularly among the urban population [36]. Inevitably, globalization of health and its indicators have triggered health system to achieve such goals including strengthening of primary health care system. These efforts were reflected in the Millennium Development Goals (MDGs) which incited a major focus on reducing disease burden and promoting good health particularly focusing on mothers and children [37]. The SDGs, which were adopted in 2016, were another historic milestone in reinforcing full focus back to health indicators which were not covered before in MDG era [38].

**Fig. 3:**
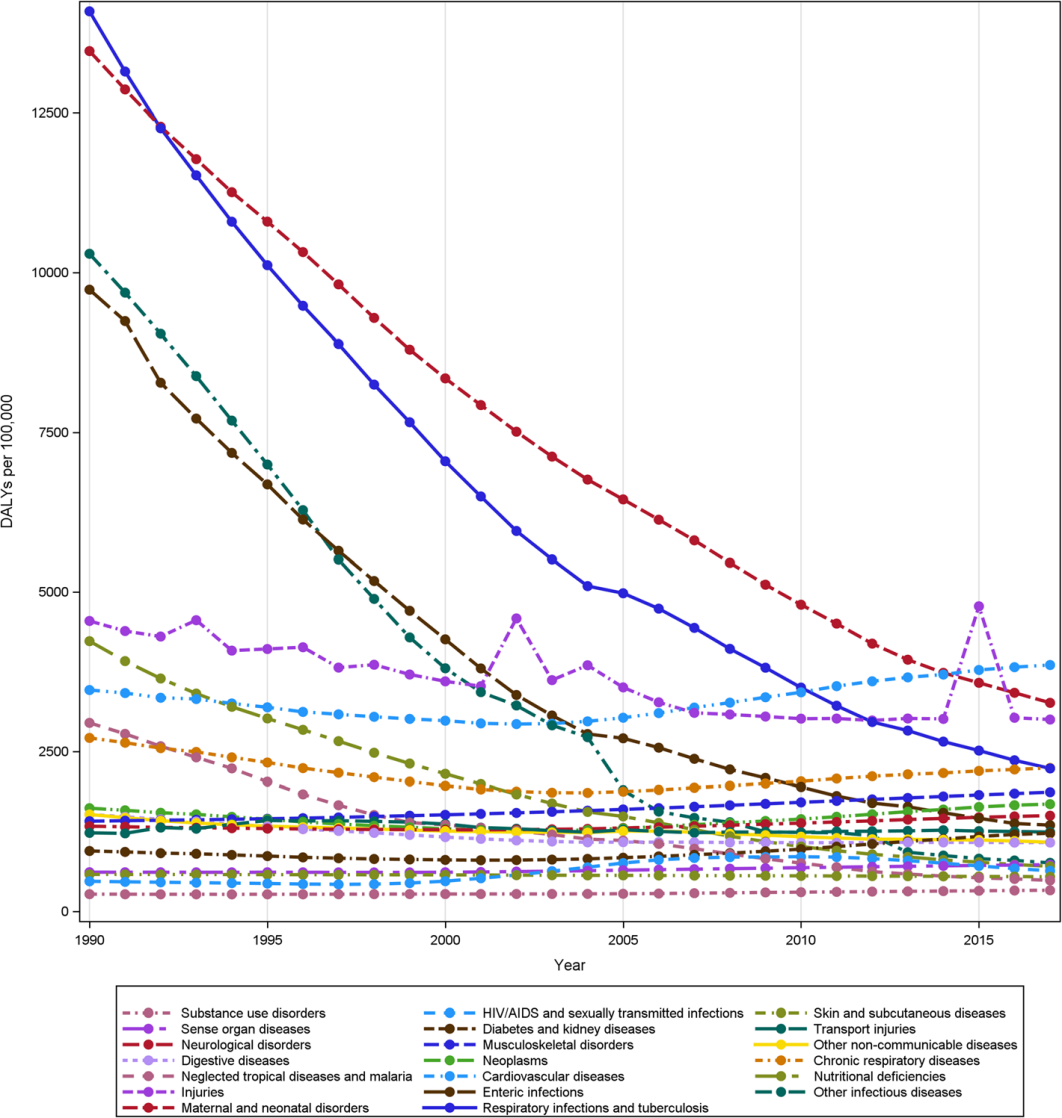
Epidemiological transition in Nepal from 1990 to 2017. The legends are ordered based on descending DALYs per 100,000 in 1990. The figure shows the shift in burden of major causes of DALYs in Nepal. Dominated by maternal and neonatal disorders, and communicable disease in 1990s—the burden of disease shifted over the next twenty years to 2017, when cardiovascular disease was the leading cause of DALYs. Data for this visualization were derived from Global Burden of Disease Study, Institute of Health Metrics and Evaluation (https://vizhub.healthdata.org/gbd-compare/)

In the follow through, the Astana Declaration called for revitalizing PHC — an agenda established 40 years ago which nudged policy makers to focus on PHC system once again. Despite significant progress in reducing the infant and maternal mortality rate, the quality of services and disparities in delivery of services were wide; and thus MDG goal 5 (improve maternal health) remained unattained [37]. For example, only 50% of women delivered their babies with the help of a skilled birth attendant and even lesser proportion of population was able to afford to and attend health centers and hospitals with significant discrepancy between rural and urban regions [37, 39]. Further, progress remained fragmented in number of areas including but not limited to non-communicable diseases (NCDs), quality of health services, and establishment of a risk pooling mechanism at the country level. In Nepal, 80% of patients attending outpatient departments present with at least one NCD and clearly demonstrates how increasing NCDs burden in Nepal aligns with the globalization of this new epidemic in Low Income and Middle Income Countries (LMICs) [27]. Based on the recent study, health service readiness towards NCDs amongst public health facilities, specifically primary health care centers in Nepal were sub-optimal and inferior compared to private hospitals in Nepal [40]. However, there has been a consistent decrease in the burden of maternal, neonatal disorders, and infectious diseases such as tuberculosis (Fig. 3). Unsurprisingly, a recent review on performance indicators of Nepal’s PHCs system urged the need to improve the coverage and quality of existing maternal, newborn and child health programs—despite these programs being around for ~ 60 years [41].

Structural challenges, including shortages in resources in the health system, will have ramifications on coverage and quality of PHC services [42]. High coverage is often equated with expansion of health services, which unfortunately, does not account for the poor performance and poor quality of existing services. Nepal’s primary and particularly rural health care system is constrained by a lack of adequate health human resources, poor supply chain logistics - medicines and equipment, attrition of health staff, their responsiveness and poor accountability [43]. Access to health services in rural regions of Nepal is additionally constrained by various barriers that includes geographic difficulties (difficult terrain, distance, poor road conditions, and lack of transportation means), and direct and indirect cost of attending health services [12, 39].

Alongside growth of PHC system in the government sector, the extensive network of private health clinics/pharmacies and hospitals emerged to become Nepal’s functional health system in urban and semi urban areas [44, 45]. REPLACE: Although, models and mechanisms of public-private partnership are critical to optimize the quality of disease specific and general health care, much of the synergies in health care are haphazard and rather compensatory to public health services [9, 40].

### Transition of health system from unitary to federal system

Nepal promulgated a new constitution in 2015 and transformed to a federal republic with seven provinces [40]. The seven provinces are sub-divided into 753 local governments comprising 6 metropolitan cities, 11 sub-metropolis, 276 urban municipal councils and 460 rural municipalities [46]. The health system in Nepal has also been restructured to align with the new federal structure.

**Fig. 4:**
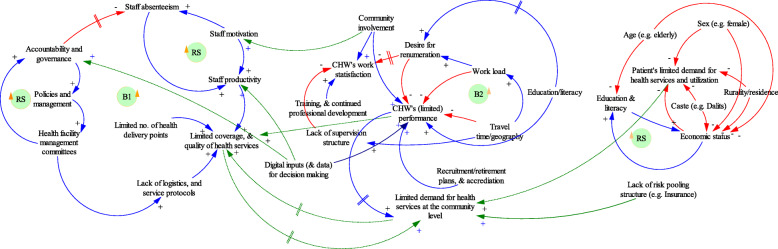
Challenges and influencing factors in Nepal’s health care system. **→ (+)** sign denotes a positive link, and **→ (−)** sign denotes a negative link where a change in influencing factor is in the same direction as the influenced element. **→ ||**indicates there is a delay where a change in influencing factor produces change in influenced element only after an interval of time. ‘RS’ denotes a reinforcing loop and ‘B’ denotes balancing loop. The figure shows the interaction of factors operating at the i) facility-level, ii) community health worker’s level (e.g. FCHVs) and iii) patients’ level in health system. Some of these factors (e.g. community involvement) operate at both the health facility and CHWs level. Development of causal loop diagrams were based on an iterative process of review incorporating author’s normative vision on health system. Thematic analysis and brainstorming with authors’ own reflexivity helped in constructing these causal loop diagrams (CLDs). More description about causal loop diagrams and its application in decision making is available in the reference [47–49]. For further information in CLDs, please follow the online resource from the John Hopkins University (https://www.coursera.org/learn/systems-thinking)

Although the federal system paved a new pathway to opportunities to building better health systems, it can suffer from pre-existing and new challenges (Fig. 4) such as inheriting structural inequities in health services (health infrastructure, geographical and economic barriers) [50, 51]. For instance, poor availability (inequitable distribution) of tertiary health institutions may continue to be major constraints for health services [45]. In addition, people in remote regions within Nepal may further suffer from geographical barriers to attending the health centres, including economic constraints in attending the health services [12]. Nonetheless, federalization that could offer local governments increased autonomy and financial independence can be an opportunity to tackle the existing challenges [9, 40]. How health is prioritized by local governments, health financing and existing constraints in human resources can be another layer to an existing challenges [50, 51]. It is essential to utilize the positive aspects of federalization to redress the challenges. Adhering to the crux of federalization that promotes local ownership, local governments must strive to plan and implement the programs [51]. Recent reports have suggested the severe constraints in local governance for health care due to inadequate health human resources [9, 52]. One way to strike a balance during the federalization process is to adopt a gradual and seamless transitioning through increased synergy between the federal and local health structure that can include integrating a feedback loop through bottom-up and community engagement approaches. The federal transition requires continued and concerted efforts through multi-stakeholder engagement to realize attainment of the SDGs [43, 53].

Reducing disparities in access to services in Nepal will further depend on the success and performance of these new federal structures—particularly whether these new structures will increase accountability of work, financial transparency, and reduce delays in implementation in exchange for devolution of power and authority [52]. The accessibility to Primary Health Care Centres (PHCCs) and the hospitals are beyond reach for a substantial proportion of the population [13], and the rural population often negotiate their rights to health care with the cost of treatment and distance to health centers [39]. For instance, only 61.8% of the households have access to health services within 30 min, with the gap between urban (85.9%) and rural (59%) being alarming [44]. While longest distance to a tertiary care centre in Kathmandu could be just 15kms away, in rural areas, a tertiary care centre could be more than 100kms away. Transition to the federal system is expected to bridge gaps in coverage of services. Nepal has already stepped into the federal structure for the last seven years, though glimmers of the functional shortcomings and gaps are emerging [9, 40] and additional evidence is likely to elucidate how these gaps can be curbed.

With the current political reformation of Nepal from unitary governance to federal governance, primary health care services have been further muddled due to a lack of clarity on devolution, responsibility distribution, and coordination [9, 54]. Poor management are reflected in various epidemic management, for instance, a recent ineffective management of Dengue fever outbreak in southern states and capital Kathmandu where a shortage of rapid diagnostic tests gained the spotlight in national and international newspapers [55]. More recently, poor preparedness and management of COVID-19 pandemic became a hallmark of globalization and highlighted the constraints in Nepal’s health system functioning including lack of coordination between federal and provincial authorities [8, 10, 56].

### Extending the risk pool for health

An effective health insurance program in a country can provide adequate financial risk protection to all of the population against the cost of health care [44]. Ensuring financial risk protection can ultimately help facilitate universal health coverage, for example by securing access to adequate health care for all at an affordable price [57]. An important step towards universal health coverage is pooling of risk in a society where all individuals share the financing of total healthcare costs, which can be channeled through either general tax revenue or by compulsory paid membership to social health insurance schemes. Successful countries with universal health coverage have adopted mixed health financing systems that protect part of the population partially through general tax revenue while others are covered by health insurance [57]. Either of these paths to universal health coverage in Nepal through health insurance scheme are jeopardized by baseline hindrances that includes inadequate health infrastructure in place and concerns around how to finance the social health scheme. In addition, countries who have achieved health coverage above 80% have strived for many years, and decades in certain instances, to continually increase coverage [57]. Amidst these pressing issues, there is a growing debate around the future structure of Nepal’s health financing mechanism. As the current roll out of the social insurance scheme has suffered pro- reach bias and low enrollment, its fate in the coming years remains uncertain. Health insurance policy in Nepal also requires alignment with the costs required to achieve the universal health coverage, including the inclusion and collaboration with private sector health services. Further research is essential to explore the costs of health care delivery and tailor the health insurance program of Nepal.

Strengthening facility-based delivery of services and improving referral mechanisms is the most frequently raised topic for improving PHC system, however, they have been inadequately prioritized amidst the fiscal and budgetary challenges. Currently, Nepal spends only 1.9% of the Gross Domestic Product (GDP) while the evidence suggests that countries should spend 5% of their GDP to progress towards universal health coverage [58, 59]. The budget on health has only marginally increased from USD 3.8 to USD 5.4 in last 19 years [58]. The current budget (484 million USD) for federal ministry of health and population of Nepal is devolved 7.4% to provincial governments and 32.2% to local governments while 60.4% remains at the central level. Around 38% of the health budget is allocated as hospital grants and 14% is allocated for capital construction. A quarter of the health budget is spent on wages and salaries. A remarkable proportion (93%) of the budget for equipment remains at the federal level, and the majority of this is allocated to purchase cancer equipment [58]. However, budget allocation is not the sole constraints; poor financial regularity, coordination between finance and managerial entities needs to be improved within the federal context, for building a value-based health care system.

### Primary health care system at crossroad

Nepal has a massive network of primary health care centers that includes health posts, and primary health care centers run by a cadre of health workers that includes doctors, nurses, paramedics and FCHVs [9]. The health system operates in four tiers with the first and basic tier composed of a massive network of the community health workers (CHWs). In this article, CHWs refer to Female Community Health Volunteers (FCHVs), who are the frontline community cadre in Nepal’s primary health care. Based on the report of 2017, there are currently 50,000 FCHVs with ratio of 20 per 10,000 populations [60]. Since its inception in 1988, the Female Community Health Volunteers’ program (FCHVs program) has been established as a crucial pillar of Nepal’s CHW program. These volunteers are selected by mother’s groups and receive 18 days of basic training on family planning, maternal, newborns, and child health and nutrition issues. Each FCHV could serve up to ~ 200 [160 to 240] community members a year depending on geographic location [61]. The second tier are primary health care centers (PHCC) that include a network of health outreach units, health posts, and primary health care centers could serve up to ~ 20,000 [16,000 to 24,000] + patients in a year. A PHCC is also a referral point (with a trained medical doctor and lab facilities) for health posts and provides training and supervision to the FCHVs. The third tier of the health care system in Nepal includes secondary-level health care facilities including former district and provincial hospitals which could serve upto ~ 100,000 [80,000 to 120,000] patients in a year [61, 62]. The fourth and the highest tier of health care system comprises tertiary-level health care facilities that consists of specialized hospitals serving upto ~ 0.66 [0.53 to 0.80] million people in a year [63]. These numbers are computed using existing information applying a generous uncertainly (±20% SD) around the estimates (visits /day) [61–63].

In these four tiers of PHC delivery system, Nepal faces a perennial shortage of skilled human resources. The World Health Organization suggests that the availability of health workers (physicians, nurses and midwives) should be ~ 45 per 100,000 population which falls short in Nepal. Current availability of health workers is 34 per 10,000 population [64, 65]. Although the vast network of primary health care has shaped up over the last decades and spans across the rural and urban region within the country, there are several challenges to the optimum functioning of such a system. In particular, the availability of human resources both in terms of quality and quantity at the community level particularly in rural areas are highly disproportionate against the rough terrain of Nepal [13].

Over the decades, the human resource gap between rural and urban regions in Nepal has not improved substantially [66] and its impacts are even more prominent during disasters [13]. During the recent 2015 earthquakes in Nepal, the rural population suffered from a disproportionate burden of morbidity and mortality, largely attributed to inadequate health services in rural regions [13, 67]. Such a huge inequity in health services is also attributed to poor management and policy, augmented by poor financial and human resource management and political instability [68, 69]. Human resources management has struggled with two primary challenges in Nepal including the lack of incentives for health human resources to serve rural regions, and second, a general lack of accountability and responsiveness of human resources working in rural regions [43, 70]. In addition, performance-based assessment, impartial treatment including the punitive actions towards irresponsive human resources and quality are critical for optimal monitoring and evaluation [71].

The challenges of management of human resources has long been felt and have achieved increasing prominence in the new federal system of Nepal [54]. In addition to the management and availability of human resources, lack of clarity in the delineation of authority between jurisdictions in the different layers of government has further impacted the federal health system. Federal governance requires an investment in development of health human resources to function under the new structure [54].

These human resource shortages have been addressed with deployment of community health workers who serve as a critical component of PHC system in Nepal [9, 40]. Assessing their strengths, and areas for improvement is an important component of strategic management – the ministry of health can leverage tools for such evaluation such as the Community Health Worker Assessment and Improvement Matrix (CHW-AIM). CHW-AIM guides policy makers and implementers through 10 core components which are considered to be essential for effective community health care systems [72]. FCHVs are the frontline staff at primary health care services in Nepal. Although much of the literature has built evidence on how FCHVs can improve health outcomes in the communities, the integration of training, and further responsibilities to FCHVs are constrained by various factors, the least of which is whether adding more responsibilities to FCHVs is feasible [71, 73]. The recently drafted FCHV strategy focuses on revitalizing the FCHV program, however, there are several loopholes which needs to be addressed to go forward. FCHVs currently lack dedicated supervisors who could provide timely mentoring and support. FCHVs need digital inputs and aids which will overcome the challenge of not having a dedicated supervisory system [74]. However, not all of Nepal’s FCHVs are literate or able to easily use a mobile device. The drafted FCHV strategy brought changes on educational and age requirement at entry and retirement that will add new cadres of more educated FCHVs into the FCHV program [75]. These are important steps towards improving community health care in Nepal, nonetheless, the effective implementation of this strategy and its output will take time.

**Fig. 5:**
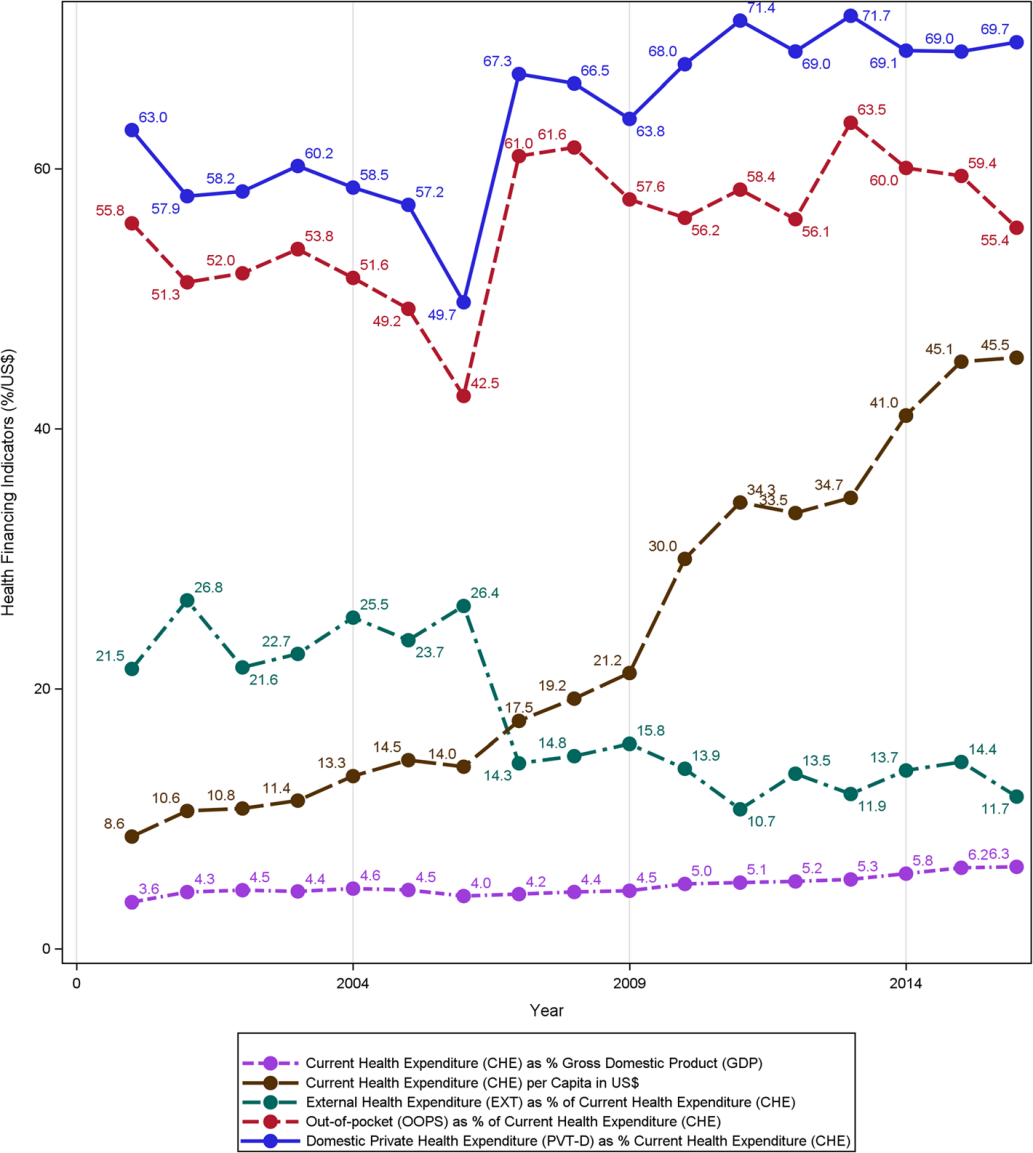
Trend in health financing indicators in Nepal from 2000 to 2016. The figure shows the changing trends of health financial indicators, notable of which is the out-of-pocket expenditure, which is above ~ 50% over past several years indicating nearly half of current health expenditure was paid out-of-pocket in these times. Data for this visualization were derived from the World Bank (https://data.worldbank.org/)

In order to strengthen and improve the network of primary health care services, alignment of policies with the current federal system is indispensable. Integrating the vertical programs with horizontal and community-based health services can help pave the way forward. Nepal’s health system inherited a challenged structure comprising vertical programs from the 1970s [76]. Going forward in the SDG era, these programs need horizontal as well as vertical integration. For instance, under horizontal integration, the NCD programs can be integrated with programs on maternal and child, and sexual and reproductive health. Under vertical integration, the community outposts such as the immunization clinics can be integrated with primary health care outreach services which can ultimately help in increasing coverage and quality of community services. Nepal’s decade long centralized approach of vertical implementation of primary health care services is characterized by inadequate horizontal implementation and community engagement [43, 77, 78]. For instance, the concept of building capacity of the frontline health staff such as female community health volunteers have recently been established to improve the clinical care including health indicators in hypertension [79, 80]. This particular window of opportunity accessible through community engagement [34, 78, 80, 81] to boost the community level health care may become promising for a new federal system of Nepal [43, 78]. Nonetheless, overriding of community health workers by adding burden to them without efforts towards 1) increasing their numbers & quality and 2) incentives can be counterproductive [82]. At current state, however, the budgetary constraints and fiscal gaps further impede the delivery of health services at the peripheral level. Figure 5 shows the trends in total health expenditure by its sources and demonstrates out of pocket expenditure as a major contributor (almost two-thirds of the health expenditure). As shown in the trend, increase in the current health expenditure but with decreasing external funded programs implies that Nepal may have to struggle to sustain the health services without external aids. Budgetary and fiscal gaps occurring in parallel with structural and policy level challenges impede the delivery of services. Similarly, the lack of public-private partnership in provision of health services, inadequate concerted efforts between non-governmental organizations and the government further misalign the agenda and may disperse the SDG goals [83, 84].

Finally, primary health care system should be built on Nepal’s long-term aspirations, particularly focusing on health, economy and development goals. The National Health Policy 2019 [85] captures the vision laid out in the Nepal’s constitution promulgated in 2015; therefore, this may provide further impetus for development in Nepal’s PHC system. Along with focus on grassroot services, strong focus on building the coverage and quality of secondary and specialist care to tackle the existing and emerging health issues (e.g., NCDs, COVID-19) is needed. Current pandemic due to COVID-19 has exposed the capacity and resilience of Nepal’s primary health care system, particularly how Nepal should garner preparedness towards the globalization of health as a major focus. A recent study that looked at health system’s preparedness towards COVID-19 management found poor coordination between three tiers of governments; inadequate delineation of responsibilities, and the least of which affected the accountability at the local levels [9]. At the heart of each of these units (mostly excluding federal level), i.e. at provincial and local levels, lack of trained health human resources affected the functionality. Echoing with the deficits that were prominent during disasters such as the 2015 Gorkha earthquakes, Nepal’s health system still needs to strive towards improving community health services by adopting community and stakeholder engagement [8, 78].

### Strengths and limitations

This review benefits from a blend of systematic search of literature, thematic synthesis of the findings which hoists to explore how globalization (targeting SDG goals, changes in economy, migration, changing disease burden including recent COVID-19 pandemic) has impact on health system’s transformation in Nepal. Nonetheless, health system and the factors affecting its performance are complex and thus findings from this study may only serve to allude towards where and why the focus is critical. The findings and its interpretations in this study further can be an initial navigation tool or launchpad for future studies around health system of Nepal. Utilizing thematic abstraction at a higher level allowed us to broaden our findings aligning with the research question, nonetheless, such an abstraction can miss the specificity of characteristics of the findings and its nuances.

## Conclusions

Nepal has been successfully improving its primary health care system since the Alma Ata declaration, and recent commitment to the Astana declaration offers important opportunity for a way forward. Globalization of health and the indicators have triggered Nepal to reform the health system, the core of which lies in improving primary health care system. This reinforces Nepal’s commitment towards strengthening the peripheral health care system and achieving the SDGs. Thus, Nepal’s health system has been transforming towards strengthening the peripheral health care networks. Globalization’s impact on political reforms, economy, labor movements and ultimately disease patterns (increasing NCDs and recent COVID-19 pandemic) have an integral and profound impact on how Nepal is transforming to tackle these challenges. With the recent political transformation from centralized vertical to federal governance with the promulgation of the new constitution in 2015, Nepal has stepped into a new era and thus requires renewed policies to overcome the challenges and materialize the gains so far. Importantly, COVID-19 pandemic further bolstered the critical role of of PHC system to deliver effective health system response. There is a clear need for health policies to adapt into the federal health system with responsibility distribution and stewardship through the municipal governments. In addition to restructuring and adapting the new health policies to suit the local governments, the existing health insurance scheme requires renewal and tailoring based on further research on costs of service delivery across the health sector to achieve the UHC quality. This may well require the multi-sectoral collaboration including public-private partnership. The vast network of community health workers/volunteers are the major assets of Nepal’s peripheral health system. These networks can be built up to increase the coverage and quality of services through an integrated approach outlined in CHW-AIM framework.

## Supplementary Information

**Additional file 1: eSupplementary material 1.** Search terms used for the review.

**Additional file 2: eSupplementary material 2.** A descriptive summary of findings from review.

## Data Availability

All data pertaining to the review are presented within the manuscript and supplementary files.
