# Supplementary material for: Transforming Nepal’s primary health care delivery system in global health era: addressing historical and current implementation challenges
Source: Global Health. 2022 Jan 31;18:8. doi: 10.1186/s12992-022-00798-5 (PMC8802254; doi:10.1186/s12992-022-00798-5)
Supplement: Supplementary file 1 — Additional file 1: eSupplementary material 1. Search terms used for the review. [file 12992_2022_798_MOESM1_ESM.docx]

# Transforming Nepal's primary health care system: addressing historical and current implementation challenges to achieve the Sustainable Development Goals

Bipin Adhikari^1,2,3*^, Shiva Raj Mishra^4^, Ryan Schwarz^5,6.7^

^1^Nepal Community Health and Development Centre, Kathmandu, Nepal

^2^Mahidol-Oxford Tropical Medicine Research Unit, Faculty of Tropical Medicine, Mahidol University, Bangkok, Thailand

^3^Centre for Tropical Medicine and Global Health, Nuffield Department of Medicine, University of Oxford, Oxford, UK

^4^Nepal Development Centre, Bharatpur, Nepal

^5^Possible, New York, NY, USA

^6^Brigham and Women’s Hospital, Department of Medicine, Division of Global Health Equity, Boston, MA, USA

^7^Harvard Medical School, Department of Medicine, Boston, MA, USA.

Corresponding author:

Bipin Adhikari, MBBS, DTM&H, MCTM, MPH, DPhil

Nepal Community Health and Development Centre, Kathmandu, Nepal and Mahidol-Oxford Tropical Medicine Research Unit, Faculty of Tropical Medicine, Mahidol University, Bangkok, Thailand

*Email: [biopion@gmail.com](mailto:biopion@gmail.com)

**eSupplementary material 1: Search terms used for the review**

Medline/PubMed: hits (n=332)

"Nepal"[MeSH] AND (("primary health care"[MeSH] OR "primary health care"[All Fields]) AND ("health system"[All Fields] OR "health system strengthening"[All Fields] OR "health system integration"[All Fields] OR "governance"[All Fields] OR "accountability"[All Fields] OR "health financing"[MeSH] OR "human resources"[All Fields] OR "supply chain"[All Fields] OR "disease control"[All Fields] OR "vertical programs"[All Fields] OR "health outcomes"[All Fields] OR "community health workers"[All Fields]) AND "humans"[MeSH Terms]

Embase: hits (n=500)

('nepal'/exp OR 'nepal'/de) AND (('primary health care'/exp OR 'primary health care'/de) AND ('health system'/exp OR 'health system'/de) OR 'health system strengthening'/exp OR 'health system strengthening'/de OR 'health system integration' OR 'governance'/exp OR 'governance'/de OR 'accountability'/exp OR 'accountability'/de OR 'health financing' OR 'human resources'/exp OR 'human resources'/de OR 'supply chain'/exp OR 'supply chain'/de OR 'disease control'/exp OR 'disease control'/de OR 'vertical programs' OR 'health outcomes'/exp OR 'health outcomes'/de OR 'community health workers'/exp OR 'community health workers'/de) AND 'human'/de

Google Scholar: hits (n=569)

Nepal "primary health care" "health system strengthening" "health system" OR "health system integration" OR governance" AND "accountability " FOR health OR financing" OR "human resources" OR "supply chain" "primary health care"
