# Supplementary material for: Transforming Nepal’s primary health care delivery system in global health era: addressing historical and current implementation challenges
Source: Global Health. 2022 Jan 31;18:8. doi: 10.1186/s12992-022-00798-5 (PMC8802254; doi:10.1186/s12992-022-00798-5)
Supplement: Supplementary file 2 — Additional file 2: eSupplementary material 2. A descriptive summary of findings from review. [file 12992_2022_798_MOESM2_ESM.docx]

# Transforming Nepal's primary health care system: addressing historical and current implementation challenges to achieve the Sustainable Development Goals

Bipin Adhikari^1,2,3*^, Shiva Raj Mishra^4^, Ryan Schwarz^5,6,7^

^1^Nepal Community Health and Development Centre, Kathmandu, Nepal

^2^Mahidol-Oxford Tropical Medicine Research Unit, Faculty of Tropical Medicine, Mahidol University, Bangkok, Thailand

^3^Centre for Tropical Medicine and Global Health, Nuffield Department of Medicine, University of Oxford, Oxford, UK

^4^Nepal Development Centre, Bharatpur, Nepal

^5^Possible, New York, NY, USA

^6^Brigham and Women’s Hospital, Department of Medicine, Division of Global Health Equity, Boston, MA, USA

^7^Harvard Medical School, Department of Medicine, Boston, MA, USA.

Corresponding author:

Bipin Adhikari, MBBS, DTM&H, MCTM, MPH, DPhil

Nepal Community Health and Development Centre, Kathmandu, Nepal and Mahidol-Oxford Tropical Medicine Research Unit, Faculty of Tropical Medicine, Mahidol University, Bangkok, Thailand

*Email: [biopion@gmail.com](mailto:biopion@gmail.com)

**eSupplementary material 3: A descriptive summary of findings from review**

| **Gaps and evidence in Nepal’s health system** | | | | | |
| --- | --- | --- | --- | --- | --- |
| **Author and year** | **Disease Focus** | **Health system context** | **Human resources** | **Medicine and equipment** | **Possible solutions** |
| Acharya 2016 [1] | Mental health | Access to psychiatrist was a major barrier in rural region of Nepal. The nearest psychiatrist was 14 hr away. | Mental health counsellors were felt to be in lower hierarchy compared to other clinicians. High staff turnover impacted patients. Reliability and access to off-site psychiatrist was a major problem. | Inappropriate usage of medications (for mental health), particularly because of lack of on-site psychiatrist. | Training primary health care providers on mental health. Recruiting/Positioning senior and junior mental health workers.  Recruiting academic psychiatrist for reliability/oversight. |
| Adhikari 2017 [2] | Disaster Medicine | Inadequate health services in rural regions, particularly for long-term impacts (e.g., mental health) due to earthquakes. | Lack of specialized health work force in the rural region, e.g., intensivist, psychiatrist | Poor reach of necessary medications and equipment affected the earthquake victims and therefore, more casualties and impacts in rural region. | Need for a policy to ensure proportionate distribution of health services to rural regions that includes, health infrastructure, specialists. Such as mobile critical care unit, mental health experts, and establishing tertiary care centres. |
| Adhikari 2015 [3] | Nursing workforce management in Nepal | Nepal’s rural health services are in constant deprivation of resources and facilities; and thus they also lack adequate incentives for nursing staff to work. The rural-urban gap in terms of health services is a huge problem, although more and more health services are flooding in urban areas. | Despite increasing number of nursing graduates in Nepal; the rural and urban nursing workforce gap continues to rise because that the policy to recruit nursing staff at rural region is ineffective in recruiting the adequate nurses and retaining them with apt incentives. This is also reflected by the discrepancies in salary/payment between government and private sector. In addition, more nursing education institutes have focused in international curriculum to ensure they are relevant for the Global Nursing jobs which means better payment. | NR | Increase the provision of incentives to serve in the rural regions including provision of career related opportunities when served at the rural regions. |
| Adhikari 2019 [4] | Generic/evaluation of free health care scheme | Free health care services had both positive and negative consequences in Nepalese population. Notably such services were not effective because of the pre-existing constraints of Nepalese health system that included service delivery was poor at the rural regions (long distance to the health centre, lack of health staff and long waiting time). The procurement and supply chain were weak leading to frequent stock out of medicine and medical equipment. | Availability of human resources were particularly poor. There was not a doctor at the rural health clinic and even if there was one appointed, his/her frequent absence made health services poor. | Medicines and medical equipment were not adequate, and its supply was not uniform. At first the free medicines were not perceived to be of good quality and second, these medicines were also not of good quality (near expiry) and some of them were unnecessarily procured, stocked and wasted. Because medicines were free, people made more demands for common medicines such as paracetamol. There were also not enough diagnostics in the rural clinics. | An overall and structural improvement in health services at rural region is critical to ensure that people utilize the free health scheme. Even if a free scheme is introduced, people felt barriers to utilize it because the health system/services suffered from pre-existing barriers. |
| Allaby 2003 [5] | Human resources for health | Role of charitable health services can be promoted and utilized within the health system. | The health services provided by the number of charitable clinics were equivalent to 330 bedded hospital in Patan. The indigenous doctors serving in these charitable clinics were altruistically motivated to serve and provide care. | Charitable clinics were devoid of good laboratory facilities despite that they had senior doctors. | To capitalise the intrinsic motivations of these charitable clinics, health planners should identify the number and type of these clinics in the area (map them) and provide subsidies for essential drugs and equipment; publicize them to make sure they are well attended, and develop a good referral network between these clinics and the mainstream health services. |
| Amano 2014 [6] | Neonatal Health | Where health system struggles to provide services, particularly due to inadequate human resources, training and utilizing community health workers can help in strengthening the health services | Female Community Health Volunteers were tasked to weigh and categorize the newborn by birth weight as normal, low-birth weight and very low-birth weight. A significant number of FCHVs could measure accurately (sensitivity of measurement was 89%). | NR | Significant number of FCHVs were able to identify LBWs and VLBWs using spring scales and shows that FCHVs can be greatly utilized including the added benefits of logistical, geographical and cultural advantages, including a high level of access to infants FCHVs have at the community. |
| Angdembe 2017 [7] | Mental health | Community health services in Nepal do not have specific services for mental health. First, the peripheral health services do not have health workers well-trained to diagnose and treat mental illnesses and second, the peripheral health workers are not allowed to prescribe psychiatric drugs by national regulatory bodies. | Lack of adequate mental health experts/psychiatrists at the peripheral health clinics or at the community and existing networks of health workers are untrained to diagnose mental illnesses and treat. Even medical officers (junior doctors) are not well-equipped to treat mental illnesses. | Lack of infra-structural facilities for mental health treatment; lack of psychiatric medicines for treatment of mental illnesses. | At first, planning to integrate mental health services in the mainstream health services is critical. Second, existing health workers could be trained to diagnose and use technologies to refer to the specialists. Third, availing of psychotropic medicines is essential through policy reforms to ensure the practitioners can use it when necessary. |
| Barker 2007 [8] | Mother and child health | The paper builds on the safe motherhood Programme (1997-2004). Safe Motherhood programme was supporting in policy-planning, service strengthening, human resources, increasing equity and access, incentives for attending the trained health workers. Rural health services suffered from lack of trained human resources; bureaucratic process made frequent transfer of the key health staff. | Safe motherhood programme suffered from the shortage of skilled professional in rural areas, especially doctors with surgical skills and experienced staff nurses; and these are aggravated by the lack of systematic planning for training, deployment and support of these key staff. | NR | The key recommendation to strengthen the health system was to develop a human resources strategy with career paths that could encourage skilled staff to stay in the government service. |
| Bhatta 2015 [9] | Telemedicine | Lack of adequate and efficient health services in rural regions is well known in Nepal. Telemedicine such as video conferencing can be a potential tool to bridge the existing gap, however there are practical challenges. | While telemedicine can be a potential solution to bridge the disproportion of health services especially in rural regions, the existing health system infrastructure and its convoluted bureaucratic process itself can impede the efficient use of telemedicine. Particularly, lack of health human resources, frequent transfers and their level of readiness to adopt such technology is pivotal in making it successful. Health workers’ motivation is key and government should have a policy to ensure they are trained and incentivized well for the technology. | Nepal has poor infrastructure and to support such technology such as irregular electricity supply, slow internet, and IT policies.  Adoption of technology needs to be constantly evolving and needs to have a budget and planning for maintenance and development. Maintenance and development of existing infrastructure have been very poor in Nepal. | Specific solutions to address the human resources problems; and IT and heath related policies are necessary to ensure the telemedicine is functional and maintained. |
| Bhattarai 2018 [10] | Optometry | Nepal has fewer than 500 eye professionals. Concentrated mostly in urban areas and are burdened with range of ocular morbidities such as cataracts, corneal ulcers and refractive errors in children rarely gets proactive assessment. | The deficiency of human resources was pilot-addressed by training school children in identifying the refractive errors of their peers and their findings were impressively sensitive and specific when compared against professional optometrists. | NR | Engaging the locally available resources such as school students to assess the refractive error possess a great potential in aiding the optometrists and health professionals, especially in rural regions and can address the health human resources gaps. |
| Byrne 2013 [11] | Review on health services related to RMNCH (Reproductive, Maternal, Newborn, and Child Health) | In rural regions of Nepal where health services are poor, it is well understood and often over emphasized that supply side has many barriers affecting the health service utilization. In fact, there are demand side barriers too that are rooted into the local social and cultural context, for example cultural reasons behind why women alone are not able to make decision to travel to a distant health clinic; case prejudice, limited knowledge of health and care, dissatisfaction with facility quality and health workers, cultural and spiritual traditions. | NR | NR | Addressing the demand side barriers is essential for example through increasing knowledge and awareness, incentives to attend the health services. Connecting with community representatives and informing consumers individually in groups and in planning sessions. Facilitating communication and transportation for patients. |
| Byrne 2014 [12] | Systematic review, RMNCH | The review draws common health system context from 11 mountainous countries that the health services suffered from rough geographical terrain, distance to health centre, lack of adequate health human resources, and their impacts on poor health services. | Community based health workers were not adequate or were not utilized in full phase. Most of the CHWs were not well trained and were not well mobilized in hard-to-reach areas. | Remote facilities are deprived of equipment and supplies in most of these countries. | The reach of services should be extended through selected, trained and supported CHWs who team with formal health workers, task-shifting to peripheral cadres, home-based administration of medications to enhance care seeking. And facilities should be equipped with supplies and staff to deliver quality, reliable and culturally sensitive care. |
| Citrin 2018 [13] | MCH/Digital database at health services | Poor rural health in Nepal also suffers from lack of database to efficiently identify the patients, their illnesses and follow-up for treatment. Study piloted a CHWs led integrated digital database construction for a routine care and follow-up of patients. | Study shows that CHWs can be provided with a digital machine to collect routine data of patients, so that the e-database can ultimately help in providing tailored treatment, population health monitoring. Also, the database can help in making an iterative design based on community/end-user engagement. | Digital equipment including fingerprint, software, and data management resources are essential. | Study has shown the integrated digital data base is an important tool to ensure the health services are effective. |
| Dawson 2008 [14] | Child health | Nepal suffers from high under-five mortality rates, low access to peripheral health facilities, and there are severe constraints in human resources. | Community based management of pneumonia by female community health volunteers with appropriate health system’s support for logistics, supervision and monitoring can bridge the constraints of human resources, and enhance the case management and prevent deaths due to childhood pneumonia. | NR | Providing training and necessary skills, and support to community-based health workers can help in improving the child health in remote communities where health services are constrained. |
| Devkota 2013 [15] | Generic/Diasporic health human resources | A high number of qualified health professionals are drained from Nepal to various countries but these health professionals form a diasporic resource capital and can be utilized if engaged well. | A systematic method to engage with the diasporic community is necessary so that we can utilize the capacity and resources that these community bear. Need for a digital directory of the diasporic health workers. | NR | At first there is a need of a mapping of diasporic community, second based on the assessment of number and qualities, a systematic engagement with the diasporic community can be initiated. The diasporic community and their resources can be utilized and formalized into Nepal’s health system through proper engagement. |
| Dhakal 2009 [16] | Generic/Health policy | Federalization of Nepal in 2008 still bears consequences at policy and its implementation. In such a context the paper explores whether and how decentralization process could be initiated and accomplished. | Study identified the challenges to the implementation of decentralization reforms as 1. Centralized and weak management and programming practices of the government; 2. Weak legal and institutional framework; 3. Conflicting policy objectives; 4. Lack of implementation strategy; 5. Poor financial and human resource management system; 6. Lack of adequate preparation for the reform; 7. Weak capacity at all levels; 8. Political instability. | NR | There needs to be adequate allocation of time and resources for preparedness to reform the policies and its implementation. Pushing for decentralization in a politically fragile environment may lead to further fragmentation of the policy and its implementation; and may be counterproductive. |
| Dulal 2014 [17] | Generic/Health system financing | The paper contextualizes the budget and financial allocation with a background changes in political system from central unitary to federal level. Also, lays out how sources of budget in Nepal are constrained. | Patients spend out of pocket to buy health in Nepal and constitutes around 60% of health expenditure. Lack of adequate budget in districts has specifically affected the logistics and cold chain maintenance for vaccines and other supplies. Lack of budget also affected the availability of drugs. The main areas of impacts by budget were on much needed human resources. Around 1/3^rd^ of the sanctioned posts for health workers remained vacant, particularly in rural region and this does not account the rise in population over the years. For instance, from 1991 to 2008, there was a 35% rise in population but unfortunately health human resources increased only by 3.4% | NR | There was a major mismatch in budget allocation and expenditure, and it highlighted how new federal structure may suffer from budgetary constraints including the pre-existing human resources problems. |
| Fredricks 2017 [18] | Disaster medicine | A huge gap in community preparedness towards disaster is well acknowledged. One of the main constraints is how Nepal has not instigated or utilized the existing cadre of community-based resources to fight the disasters. | Community based female community health volunteers (FCHVs) are critical community workforce; they are based at the community and are the frontline health staff at the community. However, during the earthquakes of 2015, these FCHVs were found to be constrained by lack of resources (trainings and health resources) to serve the population in need. Strengthening this community health workforce through training and preparedness is critical in ensuring their usefulness in future disasters. | NR | Community preparedness towards disaster can only be accomplished by strengthening the community-based health workforce (FCHVs). In future, training these FCHVs cadre is critical to ensure an effective community preparedness towards the disasters. |
| Gautam 2017 [19] | COPD | Nepal is among one of the most affected country by indoor air pollution and in recent years has been battling with increasing outdoor air pollution, all these can risk individuals to COPD. There are gaps in community-based health care in Nepal and thus COPD patients have not been identified/screened and not enough efforts have been seen in preventing the COPD. | Female community health volunteers (FCHVs) are the community cadres who could be trained to first screen the pre-COPD patients, second provide health education related to preventing indoor air pollution among others, and third can facilitate the early treatment seeking at the health centres. | Mobile phone sized spirometer could be made available at the community level for FCHVs to screen the population for COPD. | Utilization of FCHVs through training as has been echoed across the literature seem promising and could play an important role in screening and management of COPD if they are provided with the adequate resources. |
| Ghimire 2014 [20] | General Health system context | This is an opinion piece based on a medical doctor’s anecdotal experience in rural Nepal and describes accounts of poor quality of care, malpractice such as untrained health workers claiming to be specialists and quack medical practices, and how poverty has affected the rural population in attending the health services | The focus of the article is on how health care works especially qualified and trained doctors and nurses are urban centric including all the infrastructure. There is a massive gap in trained and qualified health human resources in rural region and incentivization of these health workers is necessary to ensure their retention and improvement of the care. | NR | There is a need for reform in policies guiding the health services and tertiary care centres in Nepal. Example, instead of allowing to sprout more medical colleges (tertiary care centres) in urban areas, they could be based on the region and focus on rural region. Similarly, health workers could be incentivized to serve in rural regions so that they can reduce the rural health services gap. |
| Gupta 2013 [21] | Human resources for health | Nepal suffers from poor health human resources and more so in rural region. Nonetheless, country has failed to devise a policy that could regulate the production of qualified health human resources based on the demands at the population level. | Qualified health human resources are heavily constrained in Nepal and in order to bridge the gap of poor-quality health services in rural Nepal, there is a need for a multi-stakeholder engagement in producing qualified health human resources. There is also a mismatch in absorption of the health human resources. | NR | Policies to overlook the efforts towards matching the supply and demands of health human resources is essential in Nepal. |
| Gurung 2016 [22] | Community Engagement for Health Services | Nepal suffers from poor governance that includes under-staffing, absenteeism, limited health facility opening hours, poor supervision, and monitoring, and insufficient financial management. | Increased community engagement can help these gaps by training and capacity building of local community members. This can help community reps to develop skills in co-managing community health care services | NR | Nepal’s primary health care governance could be co-managed by the community representatives. Community engagement should entail capacity building, skills development and power sharing for an impact on community led primary health care. |
| Gurung 2018 [23] | Community Engagement for Health Services | The concept of civic engagement and the existing engagement of community members with the health services is very poor in Nepal. Community’s concerns, and how their health can be improved should come from these community members. Unfortunately, community representation and its influence in the existing Nepal’s Health Facility Operation and Management Committees (HFMC) has been found to be low and of poor quality. | Community participation and engagement in health services needs to work through increasing the literacy of the population, empowering of the population for equity in health care, indiscrimination and fair representation. Community representation and inputs can make community engagement and primary health care tailored and meaningful to the local community members. | NR | Community participation just for the sake of official participation (tokenistic) has been the current scenario. There is no meaningful participation and representation of the community. Efforts towards increasing community literacy, capacity building is critical to ensure meaningful participation in future. In addition, the value of meaningful community participation should be a priority for health service providers as well. |
| Hodgins 2009 [24] | MCH | Community based primary health care is poor in Nepal and the study reports whether and after the intervention led by FCHVs helps to improve the health services. | The intervention consisted primarily of community based antenatal counselling and dispensing and an early postnatal home visit; most activities were carried out by community-based health volunteers (FCHVs). The pre-and post- evaluation showed that there were notable improvements in most household practice and service utilization indicators. | NR | The study provides how a simple intervention through community-based health workers (FCHVs) could make changes in care seeking, service utilization, and improving the health indicators. |
| Hyoju 2011 [25] | Clinical skills | Whether community-based health workers could be effectively transferred with skills necessary for breast examination that can help bring health care at the community level. | FCHVs were trained with the skills necessary to examine and screen the breasts abnormalities. FCHVs’ clinical breast examination skills to detect clinical abnormalities in comparison to the surgeons were quite good (Sensitivity: 70%, Specificity: 95%, Positive predictive value: 74%, and Negative predictive value: 94% respectively) | NR | Skills were easily transferrable to these non-technical community-based cadres and shows that it is feasible to devolve responsibilities to FCHVs for diagnosis of breast abnormalities in rural Nepal. |
| Joshi 2018 [26] | TB | Nepal’s complex geographical context and health system characteristics pose several challenges at both demand side and supply side for smooth TB treatment. The article compares facility-based DOTS versus community-based DOTS. | Traditionally TB DOTS is provided at the TB centres/facilities. Several limitations have been identified that suggest facility-based DOTS need reform. A community-based DOTS means TB medicine are made available within the community where the patient is and is provided through a community volunteer instead of patients needing to visit the health facility. | NR | Community based DOTS (CB DOTS) showed better treatment success rate (3.04% higher) and better loss to follow-up date (2.03% lower) than facility-based DOTS (FB DOTS). CB DOTS was also effective in reducing the cost by 50%. It looks like CB DOTS will be most effective for Nepal. |
| Magar 2012 [27] | Generic/Public Private Partnership | Nepal suffers from poor health services in the remote and rural region and needs immediate attention to enhance and improve the services. Both health human resources and technologies for health care are deprived in the rural region of Nepal. This is due to high urban centric medical colleges-tertiary hospitals and poor policies on PPP. | The paper describes about the possible ways to initiate and materialize the PPP between medical colleges-tertiary hospitals, their resources and the existing district level government hospitals. The PPP between the government and existing medical colleges could help foster academic activities beyond the classroom, increase disaster and epidemics preparedness, strengthen referral services, improve quality health services and increase social accountabilities of the medical colleges. | Technologies owned by medical colleges tertiary hospitals could be jointly utilized with the concept of PPP. | While there are challenges to implementing the policies for PPP, increasingly over the recent years increase in partnership between government and private entities are called for. Perhaps, modalities and spectrum of partnership can be devised so that both government and private institutions can decide to choose the level of partnership. |
| Mahato 2013 [28] | Health policy on human resource for health | Policies related to human resources and its implementation need reformation, especially based on the constraints of current policies. | Some of the policy level drawbacks include accountability of health workers in the peripheral health facilities including a robust mechanism such as reward- and punishment. There is a need to incentivize health workers to work in remote areas. There should not be a political tampering in health staff’s transfer and deployment, that can disrupt the structural integrity of the health services. Need for more health training. | NR | There is a need to review the policies and its implementation related to human resource for health. Specifically, to ensure human resource for health are adequate in remote and rural region of Nepal. |
| Marasini 2015 [29] | Generic/Public Private Partnership | Nepal is recognized to be constrained by poor resources for a quality health services and there is undoubtedly a need for public-private partnership in health sector. | PPP has helped Nepal provide good quality health services including sharing some of the health indicators as a success of the health services. However, for a robust and effective partnership there needs to be more engagement among the government staff on what is partnership, what does it constitute, and what are the outcome and indicators to look for from the partnership. This warrants a building of capacity of the government staff to ensure they can oversee the partnership. | NR | Clear policies and its implementation are essential but government staff need to understand what partnership is, how to implement it and what to expect from it rather than being a silent partner in PPP. |
| Mishra 2015 [30] | Health insurance | Also presides over the background: rural-urban gaps in health care services and why and how health insurance can address the challenges of Nepal’s poor health services and its utilization. | NR | NR | Health insurance scheme in Nepal is a new concept and needs a deliberation and careful consideration before devising the policy. While health insurance scheme may be implemented and may be accepted by the population, the prominent issues with its effective implementation lies in whether and how health system of Nepal can provide the services to those who have health insurance, particularly as Nepal suffers from poor health services in the rural and remote regions. |
| Neupane 2018 [31] | NCD | There is an increasing realization that non-communicable disease: HTN has become a major problem in Nepal. And in a context of Nepal where clinical care/health services are already stretched, screening and promoting health to prevent HTN is far-fetched. In a community randomized trial, FCHVs were trained and devolved to visit households check the blood pressure, record their dietary and life-style behaviours and provide counselling in reducing the blood pressure. Among the cohort who received the regular measurement of blood pressure and health promotion, their blood pressure was reduced compared to the control group. | The crux of the finding in this study is how community-based health care worker can be trained and devolved with the responsibilities to improve the health and wellbeing of the community members. | NR | FCHVs can be trained to provide additional service to the community and it has positive outcome such as early identification of the disease, and promotion of healthy behaviour. |
| Panday 2017 [32] | Community Health Care Workers | FCHVs form the bedrock of Nepal’s Primary Health Care. However, advocating FCHVs alone for PHC services may be an overstatement, this article explores the FCHVs challenges and limitations. | FCHVs roles and contributions in primary care in rural regions of Nepal is well acknowledged. Although less emphasized, FCHVs may have been overburdened by work/responsibilities sometimes their capacity is not enough to undertake the responsibilities. FCHVs were found to be inadequately incentivized for the roles and the work provided. |  | FCHVs roles and responsibilities may have been overburdened and they may need appropriate incentivization |
| Pant 2015 [33] | Child injuries | High number of childhood injuries are reported from rural Nepal and there are inadequate efforts or priorities towards injury prevention. | In the absence of adequate health services and human resources for community members in rural regions, female community health volunteers were trained to convene a network with the pre-existing mothers’ group to carry out injury preventive activities once they received the appropriate training. Such an injury prevention programme was successfully implemented and bears future opportunities to sustain in the rural communities. | NR | Community resources such as FCHVs and Mothers’ group are major resources but not untapped for various responsibilities, mindful of their responsibilities and ability to carry out, injury prevention can be trained and devolved to carry out in rural Nepal. |
| Regmi 2010 [34] | Health Policy | Nepal underwent federalization in 2009 and devolved power and responsibilities to local government structure. The perspectives of health workers and policy makers and impacts are essential to plan further. | Policymakers and service users seem to bear positive attitude towards the decentralization of health policy and its functioning. | NR | The qualitative study draws perceptions and aspirations from the government bodies and stakeholders during the initial stage of decentralization/federalization and is important to compare it with the current functioning of health system. |
| Schwarz 2011 [35] | Clinical care at the hospital | Remote and rural region of Nepal suffer from poor and disproportionate health care including hospitals which may have sub-standard health care services. Exploring and improving the health services of the hospital is critical. | Utilizing morbidity and mortality conference, a remote hospital in Western Nepal was able to facilitate the structured analysis of patient care and identify barriers to providing quality care. | M and M conference was guided by the following discussion items based on the Ishikawa methods that included:  **Clinical operations**—concerns with patient flow, intake, or processing in clinical departments, laboratory, radiology, or pharmaceutical operations.  **Supply-chains—**Challenges in obtaining reliable supplies of quality medicines or equipment.  **Equipment—**issues in the functioning, quality, or availability of equipment.  **Personnel—**factors pertaining to training, professionalism, management, or collaboration.  **Outreach—**issues in recruiting patients into timely and appropriate care through community engagement.  **Societal—**challenges faced by gender, caste, economic, or other social status.  **Structural—**factors related to infrastructure, such as roads, telecommunications, educational or healthcare facilities. | In resource limited settings, adapted systems-oriented M and M can be a feasible quality improvement tool. Same tool can be utilized to grade the health facilities and improve the overall health system in remote and rural regions of Nepal. |
| Schwarz 2014 [36] | Community Health Workers | FCHVs program in Nepal has been internationally applauded and has been an established frontline network for health care since 1988. It is essential to explore the FCHV program, its constraints to improve further. | Existing network of FCHV was shown to be improved by addressing and facilitating following five components that included:  1. Improve local FCHV leadership; 2. Facilitate structured weekly FCHV meetings; 3. Weekly FCHV trainings at the village level; 4. Implement a monitoring and evaluation system for FCHV patient encounters; and 5. Provide financial compensation for FCHV work. | NR | The study has shown ways to improve the existing network of FCHVs as opposed to previous studies that have relied on their responsibilities in relation to specific diseases. Five components-based activities can help improve FCHV network in future. |
| Tragard 2010 [37] | Programme integration in Health System | Global Fund funded TB, HIV and Malaria programmes in Nepal were discussed as case studies to explore the factors affecting their integration into the health system and their implementation. | TB and Malaria were well integrated in the Public Health care system, while HIV was delivered through parallel structures. Political instability, absence of continuity in leadership and inadequate investments were perceived to affect the fragmentation of financing and planning activities, especially HIV programme. There are missed opportunities to strengthen the public health system when parallel structure are in play. It is essential for public health system to be well prepared to integrate and undertake the implementation. | NR | Donor supported (including those not) programs need to be well integrated into the public health care system so that it provides opportunities to strengthen the areas to be improved and will be a learning process for the public health system in future. |
| COPD: Chronic Obstructive Pulmonary Diseases; DOTS: Directly Observed Treatment Service; FCHV: Female Community Health Volunteers; HIV: Human Immunodeficiency Virus; HTN: Hypertension; MCH: Maternal Child Health; NR=Not Reported; PPP: Public-Private Partnership; TB: RMNCH: Reproductive, Maternal, Newborn, and Child Health; PHC: Primary Health Care/Centre; TB: Tuberculosis; | | | | | |

**References**

1. Acharya B, Tenpa J, Thapa P, Gauchan B, Citrin D, Ekstrand M: **Recommendations from primary care providers for integrating mental health in a primary care system in rural Nepal.** *BMC health services research* 2016, **16:**492.

2. Adhikari B, Mishra SR, Babu Marahatta S, Kaehler N, Paudel K, Adhikari J, Raut S: **Earthquakes, Fuel Crisis, Power Outages, and Health Care in Nepal: Implications for the Future.** *Disaster Med Public Health Prep* 2017, **11:**625-632. doi: 610.1017/dmp.2016.1195. Epub 2017 Apr 1018.

3. Adhikari R: **Vacant hospitals and under-employed nurses: a qualitative study of the nursing workforce management situation in Nepal.** *Health Policy Plan* 2015, **30:**289-297. doi: 210.1093/heapol/czu1009. Epub 2014 Feb 1025.

4. Adhikari SR, Sapkota D, Thapa A, Pandey AR: **Evaluation of Nepal's Free Health Care Scheme from Health System Perspective: A Qualitative Analysis.** *J Nepal Health Res Counc* 2019, **16:**372-377.

5. Allaby MAK: **Doctors for the poor in urban Nepal.** *Tropical Doctor* 2003, **33:**83-85.

6. Amano S, Shrestha BP, Chaube SS, Higuchi M, Manandhar DS, Osrin D, Costello A, Saville N: **Effectiveness of female community health volunteers in the detection and management of low-birth-weight in Nepal.** *Rural and remote health* 2014, **14:**2508.

7. Angdembe M, Kohrt BA, Jordans M, Rimal D, Luitel NP: **Situational analysis to inform development of primary care and community-based mental health services for severe mental disorders in Nepal.** *International Journal of Mental Health Systems* 2017, **11**.

8. Barker CE, Bird CE, Pradhan A, Shakya G: **Support to the Safe Motherhood Programme in Nepal: An Integrated Approach.** *Reproductive Health Matters* 2007, **15:**81-90.

9. Bhatta R, Aryal K, Ellingsen G: **Opportunities and Challenges of a Rural-telemedicine Program in Nepal.** *J Nepal Health Res Counc* 2015, **13:**149-153.

10. Bhattarai D, Gnyawali S, Silwal A, Puri S, Shrestha A, Kunwar MB, Upadhyay MP: **Student-led screening of school children for refractive error correction.** *Ophthalmic Epidemiol* 2018, **25:**133-139. doi: 110.1080/09286586.09282017.01371767. Epub 09282017 Sep 09286522.

11. Byrne A, Hodge A, Jimenez-Soto E, Morgan A: **Looking beyond supply: a systematic literature review of demand-side barriers to health service utilization in the mountains of Nepal.** *Asia Pac J Public Health* 2013, **25:**438-451. doi: 410.1177/1010539513496139. Epub 1010539513492013 Oct 1010539513496134.

12. Byrne A, Hodge A, Jimenez-Soto E, Morgan A: **What works? Strategies to increase reproductive, maternal and child health in difficult to access mountainous locations: A systematic literature review.** *PLoS ONE* 2014, **9**.

13. Citrin D, Thapa P, Nirola I, Pandey S, Kunwar LB, Tenpa J, Acharya B, Rayamazi H, Thapa A, Maru S, et al: **Developing and deploying a community healthcare worker-driven, digitally- enabled integrated care system for municipalities in rural Nepal.** *Healthc (Amst)* 2018, **6:**197-204. doi: 110.1016/j.hjdsi.2018.1005.1002. Epub 2018 Jun 1015.

14. Dawson P, Pradhan YV, Houston R, Karki S, Poudel D, Hodgins S: **From research to national expansion: 20 Years' experience of community-based management of childhood pneumonia in Nepal.** *Bulletin of the World Health Organization* 2008, **86:**339-343.

15. Devkota A, Devkota B, Ghimire J, Mahato RK, Gupta RP, Hada A: **Involving diaspora and expatriates as human resources in the health sector in Nepal.** *J Nepal Health Res Counc* 2013, **11:**119-125.

16. Dhakal R, Ratanawijitrasin S, Srithamrongsawat S: **Addressing the challenges to health sector decentralization in Nepal: an inquiry into the policy and implementation processes.** *Nepal Medical College journal : NMCJ* 2009, **11:**152-157.

17. Dulal RK, Magar A, Karki SD, Khatiwada D, Hamal PK: **Analysis of Health Sector Budget of Nepal.** *JNMA; journal of the Nepal Medical Association* 2014, **52:**811-821.

18. Fredricks K, Dinh H, Kusi M, Yogal C, Karmacharya BM, Burke TF, Nelson BD: **Community Health Workers and Disasters: Lessons Learned from the 2015 Earthquake in Nepal.** *Prehospital and disaster medicine* 2017, **32:**604-609.

19. Gautam R, Neupane D, Karki A, Kallestrup P: **Community-based management of COPD in Nepal.** *Lancet Respir Med* 2017, **5:**e6. doi: 10.1016/S2213-2600(1016)30431-30433.

20. Ghimire P: **Nepal may have enough doctors but they're in the wrong place.** *BMJ (Online)* 2014, **349**.

21. Gupta RP, Ghimire J, Mahato RK, Kumal AB, Bc RK, Bishwakarma DK, Singh P: **Human resource for health production capacity in Nepal: a glance.** *J Nepal Health Res Counc* 2013, **11:**144-148.

22. Gurung G, Derrett S, Hill PC, Gauld R: **Governance challenges in the Nepalese primary health care system: time to focus on greater community engagement?** *Int J Health Plann Manage* 2016, **31:**167-174. doi: 110.1002/hpm.2290. Epub 2015 Mar 1030.

23. Gurung G, Derrett S, Hill PC, Gauld R: **Nepal's Health Facility Operation and Management Committees: exploring community participation and influence in the Dang district's primary care clinics.** *Primary health care research & development* 2018, **19:**492-502.

24. Hodgins S, McPherson R, Suvedi BK, Shrestha RB, Silwal RC, Ban B, Neupane S, Baqui AH: **Testing a scalable community-based approach to improve maternal and neonatal health in rural Nepal.** *Journal of Perinatology* 2010, **30:**388-395.

25. Hyoju SK, Agrawal CS, Pokhrel PK, Agrawal S: **Transfer of clinical breast examination skills to female community health volunteers in Nepal.** *Asian Pacific journal of cancer prevention : APJCP* 2011, **12:**3353-3356.

26. Joshi D, Awasthi A, Saxena A, Saxena D, Mavalankar D: **Community and facility-based tuberculosis control: Programmatic comparison and experience from Nepal.** *Clinical Epidemiology and Global Health* 2019, **7:**351-356.

27. Magar A, Subba K: **Strengthening District Health Care System through Partnership with Academic Institutions: The Social Accountability of Medical Colleges in Nepal.** *JNMA J Nepal Med Assoc* 2012, **52:**142-147.

28. Mahato RK, Ghimire J, Gupta RP: **Human resources for health in major national policies and plans of Nepal.** *J Nepal Health Res Counc* 2013, **11:**158-162.

29. Marasini B, Chaulagai Oli C, Taylor J: **Strengthening state/non-state service delivery partnerships in the health sector in Nepal.** *Int Health* 2015, **7:**228-238. doi: 210.1093/inthealth/ihv1020. Epub 2015 Apr 1015.

30. Mishra SR, Khanal P, Karki DK, Kallestrup P, Enemark U: **National health insurance policy in Nepal: challenges for implementation.** *Glob Health Action* 2015, **8:28763.:**10.3402/gha.v3408.28763. eCollection 22015.

31. Neupane D, McLachlan CS, Mishra SR, Olsen MH, Perry HB, Karki A, Kallestrup P: **Effectiveness of a lifestyle intervention led by female community health volunteers versus usual care in blood pressure reduction (COBIN): an open-label, cluster-randomised trial.** *Lancet Glob Health* 2018, **6:**e66-e73. doi: 10.1016/S2214-1109X(1017)30411-30414.

32. Panday S, Bissell P, van Teijlingen E, Simkhada P: **The contribution of female community health volunteers (FCHVs) to maternity care in Nepal: a qualitative study.** *BMC Health Serv Res* 2017, **17:**623. doi: 610.1186/s12913-12017-12567-12917.

33. Pant PR, Budhathoki B, Ellis M, Manandhar D, Deave T, Mytton J: **The feasibility of community mobilisation for child injury prevention in rural Nepal: a programme for female community health volunteers.** *BMC Public Health* 2015, **15:430.:**10.1186/s12889-12015-11783-12885.

34. Regmi K, Naidoo J, Pilkington PA, Greer A: **Decentralization and district health services in Nepal: Understanding the views of service users and service providers.** *Journal of Public Health* 2010, **32:**406-417.

35. Schwarz D, Schwarz R, Gauchan B, Andrews J, Sharma R, Karelas G, Rajbhandari R, Acharya B, Mate K, Bista A, et al: **Implementing a systems-oriented morbidity and mortality conference in remote rural Nepal for quality improvement.** *BMJ Qual Saf* 2011, **20:**1082-1088. doi: 1010.1136/bmjqs-2011-000273. Epub 002011 Sep 000226.

36. Schwarz D, Sharma R, Bashyal C, Schwarz R, Baruwal A, Karelas G, Basnet B, Khadka N, Brady J, Silver Z, et al: **Strengthening Nepal's Female Community Health Volunteer network: a qualitative study of experiences at two years.** *BMC health services research* 2014, **14:**473.

37. Trägård A, Shrestha IB: **System-wide effects of Global Fund investments in Nepal.** *Health policy and planning* 2010, **25 Suppl 1:**i58-62.
